# Lung Metastasis From Uterine Leiomyosarcoma: An Asymptomatic Presentation for a Rare Tumor

**DOI:** 10.7759/cureus.44671

**Published:** 2023-09-04

**Authors:** Parsa Tafreshi, Judy Pham, Karthik Seetharam, Tanveer Mir, Parvez Mir

**Affiliations:** 1 Internal Medicine, Wyckoff Heights Medical Center, Brooklyn, USA; 2 Cardiology, Mount Sinai Hospital, New York, USA; 3 Pulmonary Critical Care and Internal Medicine, Wyckoff Heights Medical Center, Brooklyn, USA

**Keywords:** covid-19 disease, pulmonary metastases, primary leiomyosarcoma, covid 19, uterine leiomyosarcoma

## Abstract

Uterine leiomyosarcoma (ULC) is an uncommon neoplasm characterized by poor prognosis, it can predispose to distant metastasis, causing various symptomatic presentations. We present a unique case of a large heterogeneous mass in the lung cavity arising from a ULC, with complete absence of pulmonary symptoms and with concurrent coronavirus disease 2019 (COVID-19) infection. A high degree of clinical suspicion is required for ULC with accompanying metastasis.

## Introduction

Effective screening methods are currently lacking for many cancers. Given the aggressive nature of some cancers, symptoms may indicate a certain diagnosis. However, in the absence of symptoms, cancers may be detected incidentally at an advanced stage. Here, we present the case of a patient with asymptomatic pulmonary metastasis of uterine leiomyosarcoma (ULMS).

## Case presentation

**Figure 1:**
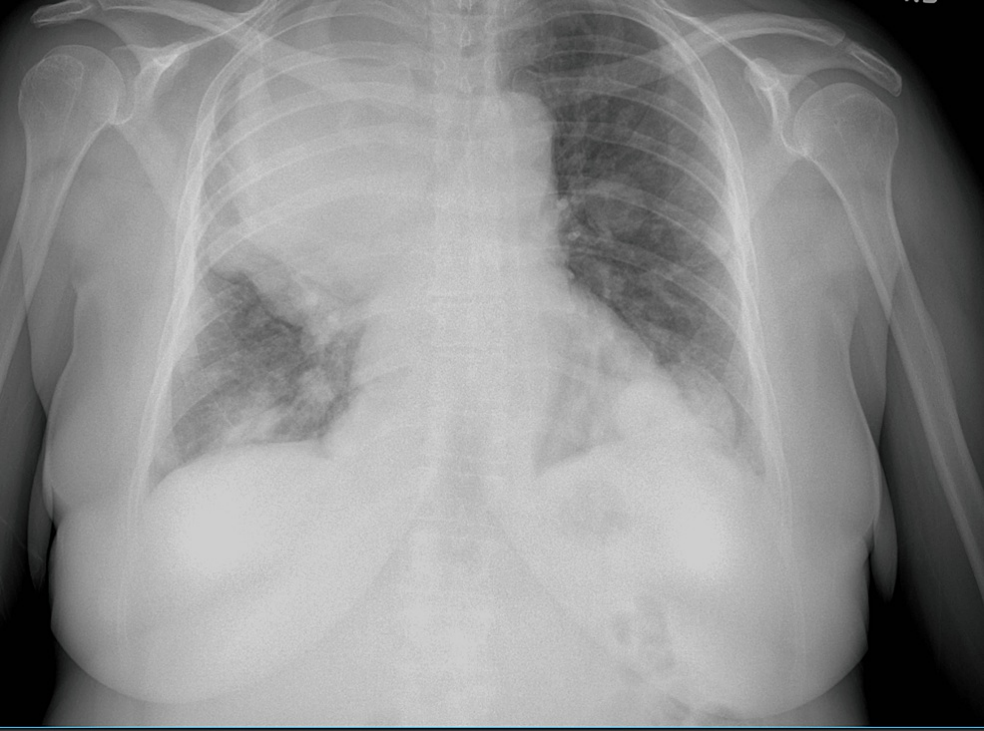
Initial CXR on admission

**Figure 2:**
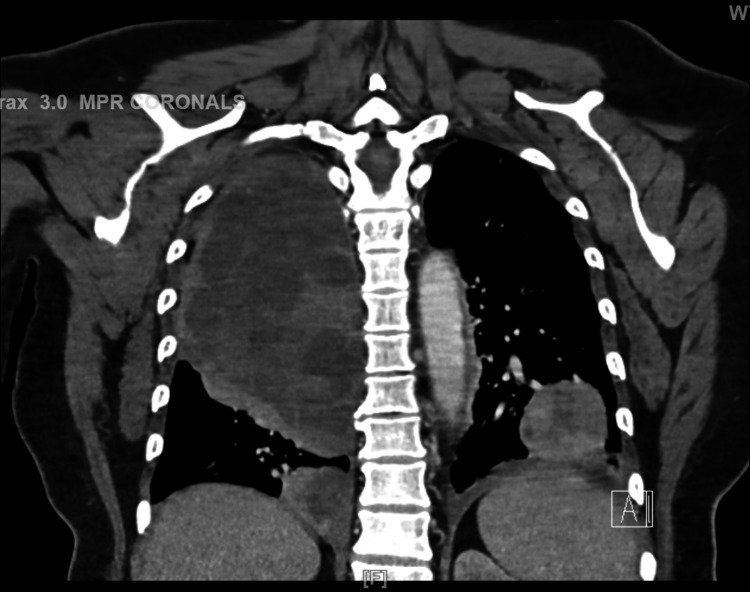
Coronal view of CT chest

**Figure 3:**
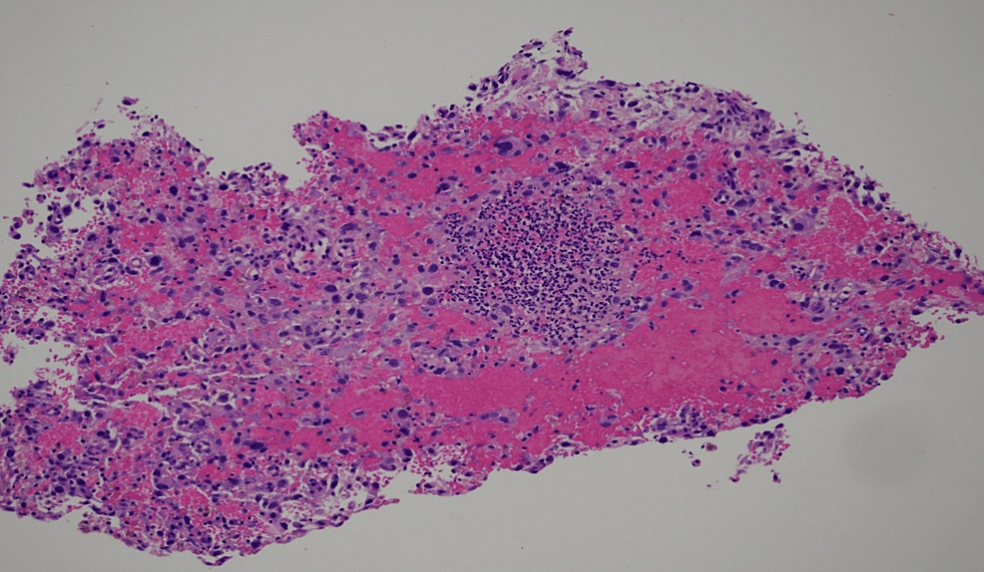
10x magnification showing blood and noncohesive tumor cells

A 52-year-old woman with a medical history of hypertension, hyperthyroidism, and fibroids presented with a one-month history of vaginal bleeding. The patient was admitted to the gynecology service for symptomatic anemia with an initial hemoglobin of 5.7 g/dL and was transfused two units of packed red blood cells. Transvaginal ultrasonography revealed a markedly enlarged heterogeneous uterus with presumed fibroids, the largest lesion possibly related to the malignancy. Follow-up computed tomography of the abdomen and pelvis with intravenous contrast revealed a markedly enlarged, irregular, and heterogeneous leiomyomatous uterus. Chest computed tomography performed to evaluate the metastasis showed a 21-cm mass in the right upper lung lobe with mediastinal and right hilar invasion as seen in Figures 1, 2. Of note, the patient was asymptomatic despite the invasive nature and size of the mass. Incidentally, the patient was also found to be positive for coronavirus disease 2019 (COVID-19). She did not require supplemental oxygen, as she maintained an oxygen saturation of 93% on room air. However, she was treated with dexamethasone 6 mg intravenous push for five days. Hematology-oncology recommended a biopsy, which was performed by interventional radiology and obtained from the lung, which confirmed the diagnosis of leiomyosarcoma. Radiation oncology recommended systemic therapy, the course of which was complicated by the COVID-19 diagnosis, as chemotherapy could not be initiated immediately. Serial imaging revealed enlargement of the mass, with a risk of airway compression. Although the patient was initially asymptomatic, she was readmitted two weeks later with a two-day history of worsening dyspnea requiring intubation. The biopsy results revealed that the patient had a ULMS with a large right upper lung lobe metastasis with discohesive malignant cells as seen in Figure 3. Given her rapid deterioration, palliative care was recommended, and per the family’s wishes, the patient was terminally extubated.

## Discussion

Leiomyosarcoma is a type of uterine sarcoma much rarer than common leiomyomas. It most commonly presents as pre- or postmenopausal bleeding. Other common symptoms of leiomyosarcomas include abdominal distention, abdominal pain, vaginal bleeding, and associated urinary symptoms [1]. If metastases are present, clues to their spread are usually observed such as dyspneic lung metastases. In our case, we saw a 21-cm mass in the right upper lung lobe that manifested no symptoms as seen in Figures 1-2. This presentation is unique, and the absence of symptoms did not correlate with the mass size. Previous studies demonstrated that this type of metastasis had a maximum size of 14 cm [2]. Furthermore, the patient had concurrent COVID-19 infection. The diagnosis of leiomyosarcoma must be made histologically from a tissue sample obtained from an endometrial, transabdominal, or transvaginal biopsy of a prolapsed mass [3]. Leiomyosarcomas are characterized by large, yellow, or tan solitary masses with soft, fleshy surfaces and areas of hemorrhage and necrosis. Histologically, these tumors exhibit cellular atypia, coagulative necrosis, and abundant mitosis. If two of these three factors are present, there is an increased risk of metastasis [4]. Discohesive malignant cells are typically observed on biopsies of the lesions as seen in Figure 3 for this patient.

At our institution, the hematology-oncology department was consulted, and a biopsy was performed. The biopsy revealed caldesmon, smooth muscle actin, CD10, and p16 positivity, whereas immunohistochemistry was negative for estrogen and progesterone receptors. P16 is overexpressed in uterine leiomyosarcoma and can be used as a diagnostic marker. The typical histological pattern of leiomyosarcoma of any origin involves intersecting sharply marginated fascicles of spindle cells with abundant eosinophilic cytoplasm and elongated hyperchromatic nuclei [5,6]. Leiomyosarcoma is a type of uterine cancer with a high risk of recurrence and death that requires rapid diagnosis and treatment to prevent mortality. Although adjuvant therapies are available for the treatment of ULMS, surgery is the cornerstone of treatment [7]. The benefits of surgical intervention in the treatment of metastatic diseases are controversial. The mass was enlarging. Several forms of adjuvant therapy have been used to treat early-stage leiomyosarcomas. These treatments include doxorubicin, docetaxel, gemcitabine, and radiation therapy [8]. Owing to the aggressive nature of leiomyosarcoma, if patients are deemed medically inoperable because they are poor surgical candidates radiotherapy can be used as a palliative option as well [9].

In our case, chemotherapy could not be attempted because of the underlying COVID-19 infection. Dexamethasone therapy was recommended for COVID-19 with lung metastasis, whereas chemotherapy was not administered. The mass enlarged and compressed the airways, and the patient was readmitted two weeks later requiring intubation. Ultimately, she was terminally extubated. This case emphasizes the importance of clinical suspicion and workup for distant metastases to the lungs in cases of ULMS despite the absence of clinical symptoms. If suspected, a full workup should be performed to evaluate the patient for the earliest possible intervention, whether surgical, adjuvant, or palliative.

## Conclusions

Leiomyosarcoma is a rare aggressive cancer that grows in the smooth muscles and requires a quick and comprehensive workup to aid in its diagnosis and treatment. Leiomyosarcoma is usually found incidentally when symptoms manifest in the patient at the most common metastatic sites such as the lung, peritoneum, bones, and liver. This case report illustrates an incidental finding of a lung mass in a patient in COVID-positive patient with no pulmonary symptoms who was later diagnosed with uterine leiomyosarcoma. Further research on ULMS in the absence or presence of minimal symptoms is required.
